# Differentiating Multiple Sclerosis From AQP4-Neuromyelitis Optica Spectrum Disorder and MOG-Antibody Disease With Imaging

**DOI:** 10.1212/WNL.0000000000201465

**Published:** 2023-01-17

**Authors:** Rosa Cortese, Ferran Prados Carrasco, Carmen Tur, Alessia Bianchi, Wallace Brownlee, Floriana De Angelis, Isabel De La Paz, Francesco Grussu, Lukas Haider, Anu Jacob, Baris Kanber, Lise Magnollay, Richard S. Nicholas, Anand Trip, Marios Yiannakas, Ahmed T. Toosy, Yael Hacohen, Frederik Barkhof, Olga Ciccarelli

**Affiliations:** From the Department of Neuroinflammation (R.C., F.P.C., C.T., A.B., W.B., F.D.A., I.D.L.P., F.G., L.H., L.M., A.T., M.Y., A.T.T., Y.H.R.C.P.C.H., F.B., O.C.), Queen Square MS Centre, UCL Queen Square Institute of Neurology, Faculty of Brain Science, University College of London; Department of Medicine (R.C.), Surgery and Neuroscience, University of Siena, Italy; Department of Medical Physics and Biomedical Engineering (F.P.C., B.K., F.B.), Centre for Medical Imaging Computing, University College of London; Universitat Oberta de Catalunya (F.P.C.), Barcelona, Spain; MS Centre of Catalonia (Cemcat) (C.T.), Vall d’Hebron Institute of Research, Spain; Radiomics Group (F.G.), Vall d’Hebron Institute of Oncology, Vall d’Hebron Barcelona Barcelona, Spain; Department of Biomedical Imaging and Image Guided Therapy (L.H.), Medical University of Vienna, Austria; NMO Clinical Service at the Walton Centre (A.J.), Liverpool, United Kingdom; Division of Multiple Sclerosis and Autoimmune Neurology (A.J.), Neurological Institute, Cleveland Clinic Abu Dhabi, United Arab Emirates; Division of Brain Sciences (R.S.N.), Department of Medicine, Imperial College London; National Institute for Health Research (NIHR) (A.T., F.B., O.C.), University College London Hospitals (UCLH), Biomedical Research Centre; and Department of Radiology and Nuclear Medicine (F.B.), Amsterdam University Medical Centre, the Netherlands.

## Abstract

**Background and Objectives:**

Relapsing-remitting multiple sclerosis (RRMS), aquaporin-4 antibody–positive neuromyelitis optica spectrum disorder (AQP4-NMOSD), and myelin oligodendrocyte glycoprotein antibody–associated disease (MOGAD) may have overlapping clinical features. There is an unmet need for imaging markers that differentiate between them when serologic testing is unavailable or ambiguous. We assessed whether imaging characteristics typical of MS discriminate RRMS from AQP4-NMOSD and MOGAD, alone and in combination.

**Methods:**

Adult, nonacute patients with RRMS, APQ4-NMOSD, and MOGAD and healthy controls were prospectively recruited at the National Hospital for Neurology and Neurosurgery (London, United Kingdom) and the Walton Centre (Liverpool, United Kingdom) between 2014 and 2019. They underwent conventional and advanced brain, cord, and optic nerve MRI and optical coherence tomography (OCT).

**Results:**

A total of 91 consecutive patients (31 RRMS, 30 APQ4-NMOSD, and 30 MOGAD) and 34 healthy controls were recruited. The most accurate measures differentiating RRMS from AQP4-NMOSD were the proportion of lesions with the central vein sign (CVS) (84% vs 33%, accuracy/specificity/sensitivity: 91/88/93%, *p* < 0.001), followed by cortical lesions (median: 2 [range: 1–14] vs 1 [0–1], accuracy/specificity/sensitivity: 84/90/77%, *p* = 0.002) and white matter lesions (mean: 39.07 [±25.8] vs 9.5 [±14], accuracy/specificity/sensitivity: 78/84/73%, *p* = 0.001). The combination of higher proportion of CVS, cortical lesions, and optic nerve magnetization transfer ratio reached the highest accuracy in distinguishing RRMS from AQP4-NMOSD (accuracy/specificity/sensitivity: 95/92/97%, *p* < 0.001). The most accurate measures favoring RRMS over MOGAD were white matter lesions (39.07 [±25.8] vs 1 [±2.3], accuracy/specificity/sensitivity: 94/94/93%, *p* = 0.006), followed by cortical lesions (2 [1–14] vs 1 [0–1], accuracy/specificity/sensitivity: 84/97/71%, *p* = 0.004), and retinal nerve fiber layer thickness (RNFL) (mean: 87.54 [±13.83] vs 75.54 [±20.33], accuracy/specificity/sensitivity: 80/79/81%, *p* = 0.009). Higher cortical lesion number combined with higher RNFL thickness best differentiated RRMS from MOGAD (accuracy/specificity/sensitivity: 84/92/77%, *p* < 0.001).

**Discussion:**

Cortical lesions, CVS, and optic nerve markers achieve a high accuracy in distinguishing RRMS from APQ4-NMOSD and MOGAD. This information may be useful in clinical practice, especially outside the acute phase and when serologic testing is ambiguous or not promptly available.

**Classification of Evidence:**

This study provides Class II evidence that selected conventional and advanced brain, cord, and optic nerve MRI and OCT markers distinguish adult patients with RRMS from AQP4-NMOSD and MOGAD.

Multiple sclerosis (MS) has a wide range of clinical and imaging manifestations, which overlap with those of neuromyelitis optica spectrum disorders (NMOSDs) and myelin oligodendrocyte glycoprotein antibody–associated disease (MOGAD).^1^ Serologic testing of aquaporin-4 (AQP4) antibody (Ab) and MOG-Ab with cell-based assays (CBAs) has high specificity.^2,3^ However, these assays are not widely available and may have variable sensitivity,^4^ leading to false-negative results.^5^ When patients are tested indiscriminately, false-positive MOG-Ab results are seen in 28% of cases.^6^ In addition, antibody levels may fluctuate, and outside an acute event they may be negative in up to 57% of patients with MOGAD^7^ and decline up to become negative in the remission phase of NMOSD.^8,9^ When serologic testing is unavailable or ambiguous, or a false-negative serologic result is suspected, MRI can be of value to support the differential diagnosis.

Differences in patterns of brain and spinal cord lesions between relapsing-remitting MS (RRMS), AQP4 antibody–positive neuromyelitis optica spectrum disorder (AQP4-NMOSD), and MOGAD have been described.^10,11^ In RRMS, white matter lesions tend to affect specific brain regions, such as the periventricular and juxtacortical white matter, the corpus callosum, and the infratentorial areas,^12^ whereas in AQP4-NMOSD, brain abnormalities are frequently located in areas with high AQP4 expression (e.g., periependymal lesions surrounding the ventricles or involving corticospinal tracts).^13^ In adult MOGAD, brain MRI can be unremarkable or show large, ill-defined or defined lesions, mostly located in the deep gray matter and the cerebellar peduncles.^14^ Longitudinally extensive transverse myelitis is the hallmark of AQP4-NMOSD with predilection for the cervical cord, whereas in MS, multiple, short-segment lesions are common, mostly located in the cervical cord. In MOGAD, cord lesions often affect the lower thoracic cord and conus and tend to be longitudinally extensive in the acute stage.^15^ Imaging features, which are very suggestive of a specific disease, may not be seen anymore in the nonacute phase; this is common in patients with MOGAD.^16^ In addition, the approach of reaching a diagnosis of 1 of these 3 diseases on the basis of typical MRI features alone (or in combination) is not standardized.^17^

With regard to advanced MRI markers, cortical lesions are well described as distinctive features of MS,^18^ whereas they are rarely seen in AQP4-NMOSD and MOGAD.^19,20^ The central vein sign (CVS) is detectable in a higher percentage of brain lesions in RRMS than AQP4-NMOSD^21^ and MOGAD.^22^ Gray matter atrophy is seen in MS, but not in NMOSD^23^; it is unknown whether gray matter volumes distinguish between RRMS and MOGAD. Previous studies showed a greater cervical cord atrophy in AQP4-NMOSD than in RRMS, but no cord atrophy was detected in MOGAD.^24,25^ Although microstructural damage of the cord in RRMS and AQP4-NMOSD was found using diffusion tensor imaging (DTI), no substantial changes were detected in MOGAD.^25^

Optic neuritis is a common feature of these 3 diseases. In RRMS, optic nerve lesions on orbital MRI are often unilateral, short, and anterior, whereas in AQP4-NMOSD and MOGAD, they are mostly bilateral and long, although posterior in the former and anterior in the latter.^26^ Optic nerve atrophy and microstructural damage can be detected with quantitative MRI techniques.^27^ Magnetization transfer ratio (MTR) of the optic nerve in the different segments of patients with NMOSD has not been assessed, whereas studies in MS showed no definitive results.^28,29^ Optical coherence tomography (OCT)^30^ has been widely used in MS, demonstrating a thinner retinal nerve fiber layer (RNFL) in AQP4-NMOSD than MS,^31^ while showing conflicting results when comparing the 3 diseases.^32^ It is unknown whether the inclusion of optic nerve markers might improve the differentiation between MS and the 2 antibody-mediated diseases in the nonacute phase.

The primary research question of this study is to identify selected conventional and advanced brain, cord, and optic nerve MRI and OCT markers to distinguish adult patients with RRMS from APQ4-NMOSD and MOGAD. We investigated whether MRI characteristics, known to be typical of MS, discriminate between RRMS and the 2 antibody-mediated diseases alone and in combination and whether including optic nerve imaging measures may enhance the accuracy of the discrimination.

## Methods

### Subjects

Patients older than 18 years with a diagnosis of (1) RRMS according to the 2017 McDonald criteria,^1^ (2) AQP4-NMOSD according to the Wingerchuk criteria,^33^ or (3) MOGAD (defined as MOG-Ab positivity using CBAs in the context of an acute demyelinating event in patients presenting with a MOGAD phenotype previously described^34^), seen at the National Hospital for Neurology and Neurosurgery, London, and the NMO Clinical Service at the Walton Centre, Liverpool, between 2014 and 2019, were recruited consecutively. Antibody testing using either live or fixed CBA was performed as part of the clinical evaluation in the local, clinical laboratories. The threshold for serum MOG-Ab CBA positivity was immunoglobulin G1 at 1:20, followed by 1:200 for H&L secondary antibody. To avoid the inclusion of false positives, only patients with a secure positivity without low or borderline autoantibodies results were included. Age- and sex-matched healthy controls were also recruited. Participants were excluded if they had major contraindications to MRI, a neurologic comorbidity, any ophthalmic conditions (such as glaucoma, ocular trauma, or degenerative eye disease), or a relapse in the previous 6 months. Data from a subgroup of these patients have been previously reported.^21^

### Clinical Assessment and OCT

At the time of the MRI, patients' disability was assessed using the Expanded Disability Status Scale (EDSS), the timed 25-foot walk test (TWT), the 9-hole peg test (9-HPT), and the Symbol Digit Modalities Test.^35^ Visual assessments for each eye were performed using high-contrast letter acuity (VA100%) with a retroilluminated Early Treatment Diabetic Retinopathy Study chart at 4 m, and low-contrast letter acuity with a retroilluminated 2.5%, and 1.25% Sloan charts.

Patients and controls underwent peripapillary RNFL and macular volume OCT scanning using Heidelberg Eye Explore 1.10.2.0 (Spectralis version 6.9a, Heidelberg Engineering, Heidelberg, Germany). Peripapillary RNFL and macular ganglion cell–inner plexiform layer (GCIPL) thickness were extracted. A quality check was performed according to the international OSCAR-IB criteria.^36^

### MRI Data Acquisition and Analysis

All participants underwent a 3T MRI scan at the Queen Square MS Centre, London, using a 32-channel head coil (see protocol details in eTable 1, links.lww.com/WNL/C402). Brain T2 lesions were semi-automatically segmented using JIM v.6.0, whereas cervical cord lesions were manually identified on sagittal T2-weighted and axial FFE scans.

For brain tissue parcellation, we used the geodesic information flows method,^37^ after an automated T1 brain lesion–filling technique.^38^ The fractional volumes of whole brain, white matter, gray matter, and deep gray matter relative to total intracranial volume were calculated. Cortical lesions were manually identified on Phase Sensitive Inversion Recovery images and scored as leukocortical or intracortical^39^ by consensus between 2 raters (R.C. and L.H.) and a senior neuroradiologist (F.B.), who reviewed the cases of disagreement.

For the CVS analysis, the T2-weighted images were affine coregistered to the susceptibility-weighted imaging (SWI) using a symmetric and inverse-consistent approach. The identification of the CVS (indicating the presence of a central vessel, predominantly veins and venules, in MS plaques) was obtained on SWI with the fully blinded analysis previously described^21^ and following the NAIMS criteria.^40^ The proportion of lesions with the CVS out of the total number of lesions was reported. The presence of the CVS was based on the consensus between 2 raters (R.C. and L.H.). The mean cross-sectional area (CSA) of the cord was calculated at C2-C3, using the active surface model (JIM v.6.0).^41^

Diffusion-weighted images were processed using FMRIB Software Library and the SCT (FMRIB Software Library v.0.5).^42,43^ The mean values of diffusion metrics within the whole cord were calculated (eFigure 1, links.lww.com/WNL/C402). Magnetization transfer imaging acquisition was performed separately for each eye; the mean MTR values in the whole optic nerve and in the intraorbital, intracanicular, intracranial segments were obtained (eFigure 2, links.lww.com/WNL/C402).

Raters worked independently, blinded to clinical data; they had a good interrater agreement (Cohen kappa coefficients ≥92%). During the study, a major MRI system upgrade took place (new scanner software, from release 3 to 5; new hardware, from Philips Achieva to Ingenia-CX), which was considered in the statistical analysis.

### Statistical Analysis

Age, sex, clinical, and lesion characteristics were compared between RRMS, AQP4-NMOSD, MOGAD, and healthy control groups using the χ^2^ test, linear regression, Mann-Whitney *U* tests, or mixed-effect regression models, depending on the nature of the variable.

The analyses for this study were then divided into the following 2 parts:

1. Differences in brain, cervical cord, and optic nerve measures between diseases and their association with clinical measures

Multiple linear regression models were fitted to evaluate differences in brain and cord MRI metrics between groups and their associations with clinical measures. The following analyses were performed: (1) estimation of differences in brain and cord MRI measures (lesions, brain parenchymal fraction, white matter fraction, gray matter fraction, deep gray matter fraction, CSA, and DTI metrics) between the 3 patient groups and controls, where MRI measures were the dependent variables and patient group the explanatory variable, and (2) assessment of correlations between MRI metrics, and clinical measures in each patient group separately, where clinical measures were the dependent variables (one at a time) and MRI metrics the explanatory variables.

Random-intercept mixed-effects regression models were used to assess differences between patient groups in optic nerve metrics (visual acuity, average RNFL and GCIPL thickness, and average MTR of the whole optic nerve and each segment) between patient groups and between patients and controls, with a group indicator as the main covariate. Multiple mixed-effect regressions were used to assess correlations between optic nerve metrics different between patients and controls and clinical measures in each group. These models enabled us to perform the analyses considering that the observations corresponding to each pair of eyes were correlated and belonged to the same subject.

2. Identifying imaging markers that discriminate between diseases

To identify the MRI and OCT variables discriminating between diseases, the variables that showed significant differences between any disease group pair were entered into forward stepwise logistic regression models. First, we ran univariable logistic regression analyses, with patient group as the dependent variable and MRI measures as covariates, one at a time. For optic nerve measures, the average between the 2 eyes was used. To select the best set of predictors, each imaging measure was added individually to a model already adjusted for age, sex, and upgrade. If these imaging measures one at a time were significant, were kept for the next stage, added sequentially to the basic model and kept if significant. The order of this addition was determined by the individual accuracy of the measures. If 2 variables had individually the same accuracy, the variable with the lowest Bayesian information criterion was chosen first. From all models, we obtained the OR of having one disease vs another (i.e., RRMS vs AQP4-NMOSD, RRMS vs MOGAD, and AQP4-NMOSD vs MOGAD), the accuracy, and the area under the curve (AUC) receiver operating characteristic curve.

In each group, for each imaging predictor, the best cutoff (i.e., the value associated with the highest accuracy) that predicted the outcome (e.g., a diagnosis of RRMS vs AQP4-NMOSD or MOGAD) was calculated.

All the analyses were corrected for age, sex, and upgrade of the scanner. Other potential confounders, such as disease duration, presence of brain or cervical cord lesions and atrophy measures in the brain and the spinal cord, and number of optic neuritis, were also considered, as appropriate.

Analyses were performed using Stata 15.1 software (Stata Corporation, College Station, TX, USA). Statistical significance was considered when *p* values were <0.01.

### Data Availability

Anonymized data not published within this article will be made available by request from any qualified investigator.

### Standard Protocol Approvals, Registrations, and Patient Consents

Written informed consent was obtained from all participants. The study was approved by the National Research Ethics Service Committee London Bloomsbury and complied with the Data Protection Act 2018.

## Results

### Participant Characteristics

A total of 91 patients (31 RRMS, 30 AQP4-NMOSD, and 30 MOGAD) and 34 healthy controls were included in the study (the flowchart of patients is given in eFigure 3, links.lww.com/WNL/C402). Thirty (100%) patients with AQP4-NMOSD and 25 (83%) patients with MOGAD were tested using live CBAs, whereas the remaining using fixed assays. Patients with AQP4-NMOSD had the highest EDSS score and the worst high- and low-contrast visual acuity, whereas patients with MOGAD were the youngest and had the shortest disease duration (all *p* < 0.001). A relapsing disease course was reported in 87% patients with AQP4-NMOSD and 67% patients with MOGAD. The most common clinical presentations at onset in the 2 antibody-mediated diseases were optic neuritis and transverse myelitis (Table 1). Details about MOG-Ab testing timing are provided in eTable 2 (links.lww.com/WNL/C402).

**Table 1:**
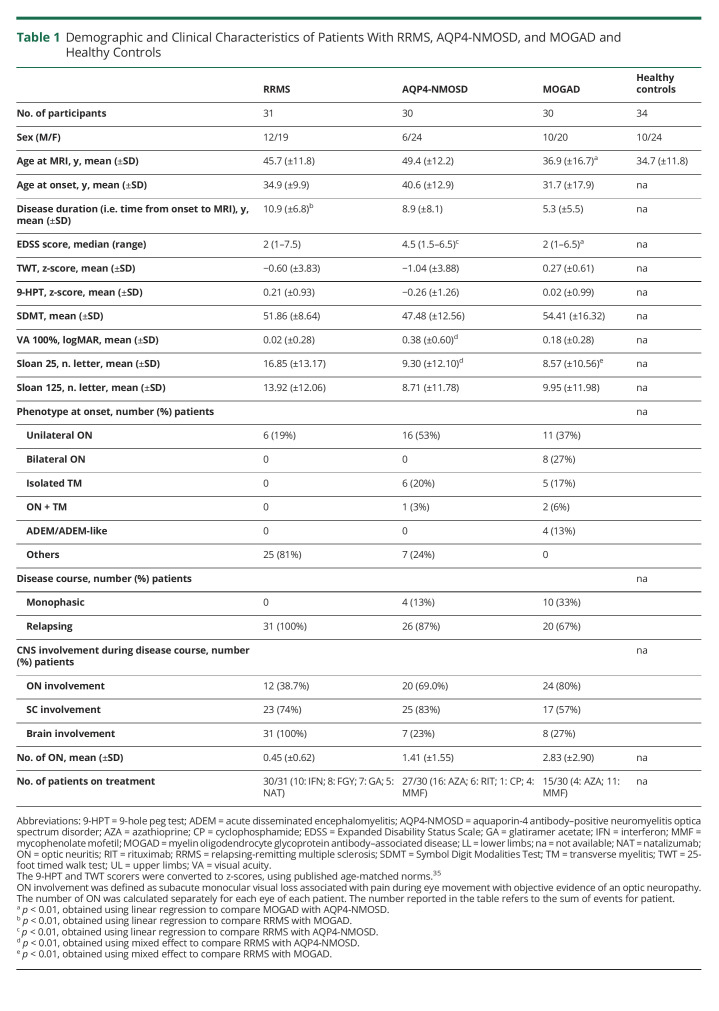
Demographic and Clinical Characteristics of Patients With RRMS, AQP4-NMOSD, and MOGAD and Healthy Controls

### Differences in Brain, Cervical Cord, and Optic Nerve MRI and OCT Measures Between the 3 Diseases

**Figure 1:**
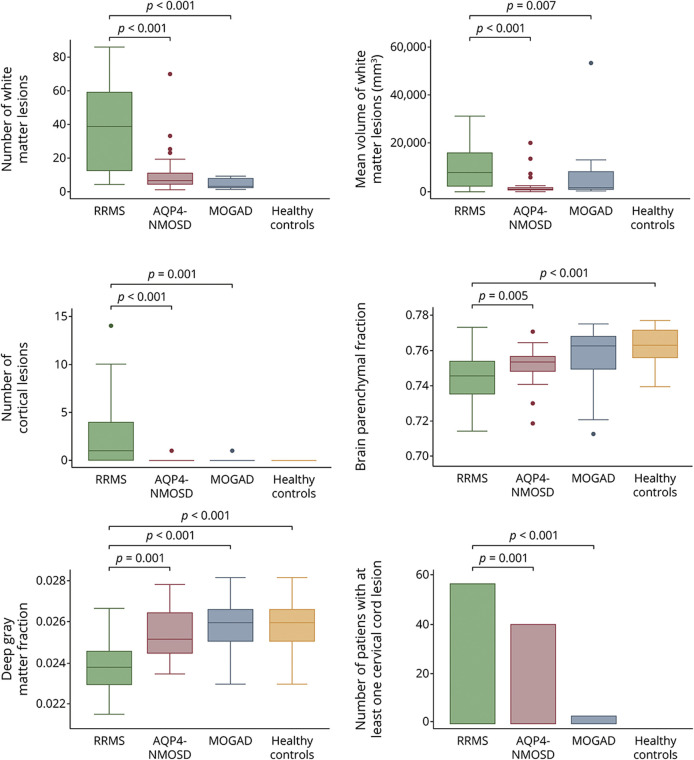
Differences in Brain and Cervical Cord Measures Between RRMS, AQP4-NMOSD, MOGAD, and Healthy Controls The boxplots show a lower number and volume of lesions and a higher degree of atrophy in patients with RRMS than patients with AQP4-NMOSD and MOGAD and healthy controls. AQP4-NMOSD = aquaporin-4 antibody–positive neuromyelitis optica spectrum disorder; MOGAD = myelin oligodendrocyte glycoprotein antibody–associated disease; RRMS = relapsing-remitting multiple sclerosis.

Differences between diseases are summarized in Table 2. Brain white matter lesions were detected in 100% of patients with RRMS, 83% of patients with AQP4-NMOSD, and 27% of patients with MOGAD. The mean number and volume of lesions were higher in RRMS than AQP4-NMOSD (*p* < 0.001) and MOGAD (*p* < 0.001 and *p* = 0.007). No difference in the brain lesion number or volume between AQP4-NMOSD and MOGAD was identified (Figure 1). The presence of at least 1 cervical cord lesion was more common in RRMS (55% of the cases) than AQP4-NMOSD (40%) and MOGAD (4%) (*p* < 0.001).

**Table 2:**
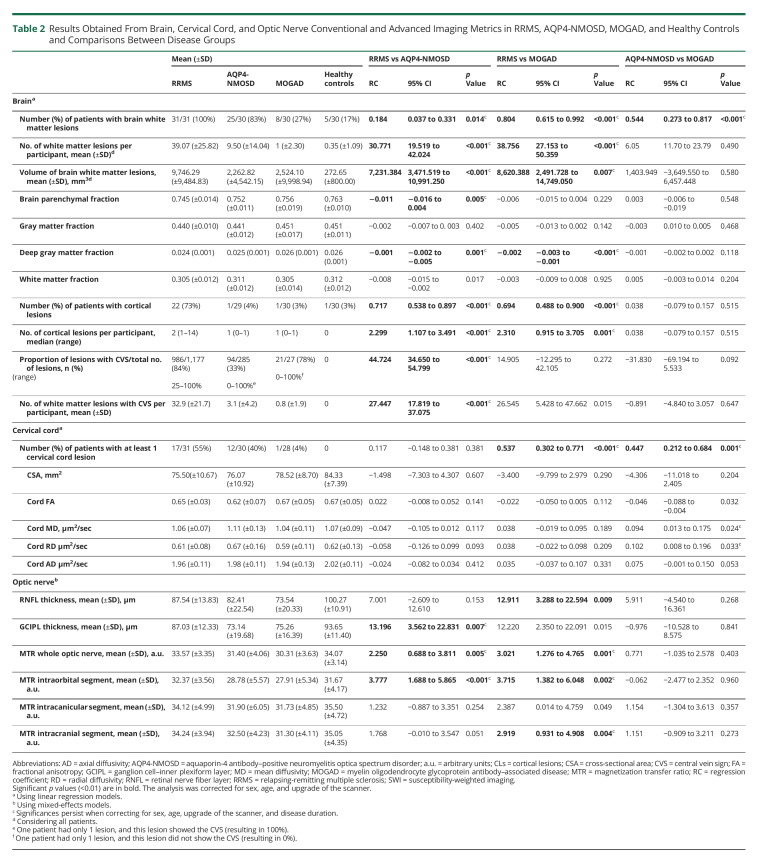
Results Obtained From Brain, Cervical Cord, and Optic Nerve Conventional and Advanced Imaging Metrics in RRMS, AQP4-NMOSD, MOGAD, and Healthy Controls and Comparisons Between Disease Groups

Patients with RRMS showed lower brain parenchymal fraction, white matter fraction, and deep gray matter fraction than healthy controls (*p* < 0.001, *p* = 0.009, and *p* < 0.001, respectively), lower brain parenchymal fraction and deep gray matter fraction than AQP4-NMOSD (*p* = 0.005 and *p* = 0.001, respectively), and lower deep gray matter fraction than MOGAD (*p* < 0.001). Patients with MOGAD did not differ from healthy controls and from AQP4-NMOSD.

**Figure 2:**
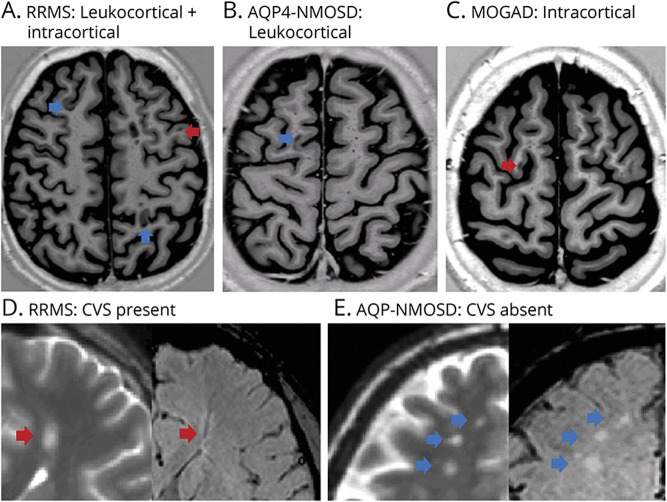
Examples of Cortical Lesions Seen on Phase-Sensitive Inversion Recovery (PSIR) Images and Lesions With and Without the Central Vein Sign (CVS) on Susceptibility-Weighted Imaging (SWI) in RRMS, AQP4-NMOSD, and MOGAD In the upper figures, PSIR imaging showing lesions located exclusively in the cortex (intracortical, red arrow) or within the cortex and adjacent juxtacortical white matter (leukocortical, blue arrow) in RRMS, AQP4-NMOSD, and MOGAD. Intracortical and leukocortical lesions were detected in patients with RRMS (A), whereas 1 leukocortical lesion in 1 patient with AQP4-NMOSD (B) and 1 intracortical lesion in 1 patient with MOGAD (C) were found. In the lower figures, T2 and corresponding SWI of deep white matter lesions with (red arrow) or without (blue arrow) CVS in RRMS and AQP4-NMOSD. The dark vein was located centrally in a lesion in an RRMS patient (D), while it was absent in three lesions in an AQP4-NMOSD patient (E). AQP4-NMOSD = aquaporin-4 antibody–positive neuromyelitis optica spectrum disorder; MOGAD = myelin oligodendrocyte glycoprotein antibody–associated disease; RRMS = relapsing-remitting multiple sclerosis.

Cortical lesions were detected in 73% of patients with MS, 4% of patients with AQP4-NMOSD, and 3% of patients with MOGAD. There were a higher number of cortical lesions in RRMS (total of 74: 40 leukocortical and 34 intracortical, with a median of 2 lesions per patient) than AQP4-NMOSD (only 1 leukocortical lesion in 1 patient) and MOGAD (only 1 intracortical lesion in 1 patient) (Figure 2).

**Figure 3:**
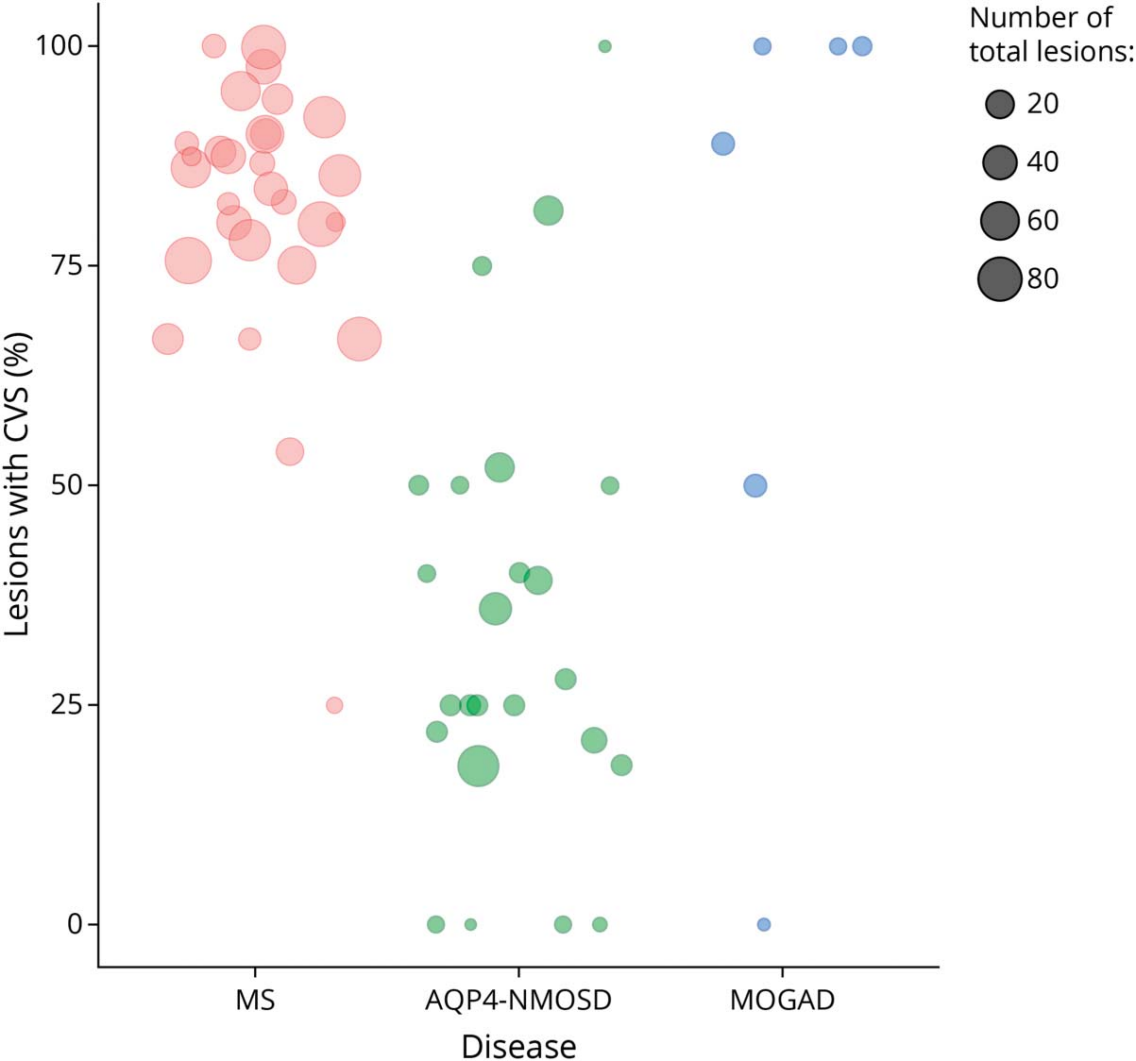
Central Vein Sign (CVS) Detected on Susceptibility-Weighted Imaging (SWI) in the Patient Groups The scatterplot shows the proportion of lesions with CVS (out of the total number of lesions) for each patient in the 3 diseases (orange = RRMS, green = AQP4-NMOSD, and blue = MOGAD). Patients without brain lesions are not displayed. Two of 8 patients with MOGAD were excluded from the rating (1 for poor SWI quality and 1 for extensive, confluent PD/T2 abnormalities). Note: 100% means that all lesions assessed showed the CVS. MOGAD = myelin oligodendrocyte glycoprotein antibody–associated disease; RRMS = relapsing-remitting multiple sclerosis.

The CVS within white matter lesions on SWI was observed in 100% of patients with RRMS, 70% of patients with AQP4-NMOSD, and 17% of patients with MOGAD. The proportion of lesions with the CVS was higher in RRMS (84%) than AQP4-NMOSD (33%) but did not differ between AQP4-NMOSD and MOGAD (Figures 2 and 3).

Both patients with RRMS and AQP4-NMOSD showed smaller cervical cord CSA than healthy controls (*p* = 0.001 and *p* = 0.003, respectively), whereas patients with MOGAD did not show cervical cord atrophy. Patients with AQP4-NMOSD showed lower fractional anisotropy (FA) than healthy controls (regression coefficient [RC]: −0.043, 95% CI: −0.71 to −0.014, *p* = 0.003). No differences were found between RRMS and MOGAD and healthy controls and between the 3 diseases.

All patient groups showed lower RNFL thickness than healthy controls, with the 2 antibody-mediated diseases also showing lower GCIPL than healthy controls (all *p* < 0.01) (eFigure 4, links.lww.com/WNL/C402). When compared with RRMS, GCIPL thickness was lower in AQP4-NMOSD (*p* = 0.007), whereas RNFL thickness was lower in MOGAD (*p* = 0.009). Patients with AQP4-NMOSD and MOGAD showed lower average MTR of the whole optic nerve and the intraorbital segment compared with RRMS and healthy controls (all *p* < 0.01). MOGAD showed lower MTR of the intracranial segments when compared with RRMS and healthy controls. No differences in OCT and optic nerve MTR indices were found between the 2 antibody-mediated diseases.

### Association Between Clinical Measures and Imaging Measures

In RRMS, worse 9-HPT z-score was associated with lower brain white matter fraction (RC: 0.07, 95% CI: 0.02 to 0.15, *p* = 0.005) and lower CSA (RC: 0.04, 95% CI: 0.01 to 0.07, *p* = 0.007), and worse high-contrast VA was associated with reduced RNFL thickness (RC: −0.01, 95% CI: −0.02 to −0.004, *p* = 0.001). In AQP4-NMOSD, worse EDSS score and TWT z-score were associated with lower cord CSA (RC: −0.08, 95% CI: −0.14 to −0.03, *p* = 0.006; RC: 0.24, 95% CI: 0.13 to 0.34, *p* < 0.001, respectively), and worse 9-HPT and greater vibration dysfunction with lower cervical cord FA (RC: 11.74, 95% CI: 6.19 to 17.28, *p* < 0.001; RC: −72.31, 95% CI: −102.48 to −42.15, *p* < 0.001, respectively). Worse high-contrast VA was associated with lower average MTR of the whole optic nerve and the intraorbital segment (RC: −0.07, 95% CI: −0.10 to −0.03, *p* < 0.001; RC: −0,04, 95% CI: −0.07 to −0.02, *p* = 0.002, respectively).

In MOGAD, worse high-contrast VA was associated with reduced RNFL thickness (RC: −0.004, 95% CI: −0.07 to −0.001, *p* = 0.003), reduced GCIPL thickness (RC: −0.006, CI: −0.009 to −0.002, *p* = 0.002), and lower average MTR of the whole optic nerve and the intraorbital segment (RC: −0.03, 95% CI: −0.05 to −0.01, *p* = 0.001; RC: −0,03, 95% CI: −0.04 to −0.01, *p* < 0.001, respectively).

### MRI and OCT Discriminators Between the 3 Diseases

#### RRMS vs AQP4-NMOSD

The proportion of lesions with the CVS was the most accurate measure that distinguished RRMS from AQP4-NMOSD (OR: 1.09, 95% CI: 1.05–1.14, accuracy: 91%, specificity: 88%, sensitivity: 93%, AUC: 0.93, *p* < 0.001). This means that for each percentage unit of increase in the proportion of lesions with CVS, there was a 9% increased risk of having RRMS instead of AQP4-NMOSD. The best cutoff value that predicted RRMS was a proportion of lesions with CVS of 54%.

The second most accurate discriminator was cortical lesion number (OR: 32.52, 95% CI: 3.52–300.03, accuracy: 84%, specificity: 90%, sensitivity: 77%, AUC: 0.91, *p* = 0.002), followed by brain white matter lesion number (OR: 1.07, 95% CI: 1.03–1.11, accuracy: 78%, specificity: 84%, sensitivity: 73%, AUC: 0.85, *p* = 0.001), and deep gray matter fraction (OR: 0.48, 95% CI: 0.30–0.78, accuracy: 76%, specificity: 79%, sensitivity: 73%, AUC: 0.80, *p* = 0.003). The best cutoff values that predicted RRMS were a number of cortical lesions of 1 and of brain white matter lesion of 11.

The last 2 significant discriminators were the brain parenchymal fraction (OR: 0.48, 95% CI: 0.28–0.83, accuracy: 66%, specificity: 72%, sensitivity: 60%, AUC: 0.76, *p* = 0.008) and the optic nerve MTR (OR: 1.32, 95% CI: 1.04–1.68, accuracy: 66%, specificity: 60%, sensitivity: 71%, AUC: 0.73, *p* = 0.023). In a multivariable model, the combination of higher proportion of lesions with CVS, higher number of cortical lesions, and higher average MTR of the whole optic nerve achieved the highest accuracy in indicating a diagnosis of RRMS rather than AQP4-NMOSD (accuracy: 95%, specificity: 92%, sensitivity: 97%, AUC: 0.97, *p* < 0.001) (Table 3).

**Table 3:**
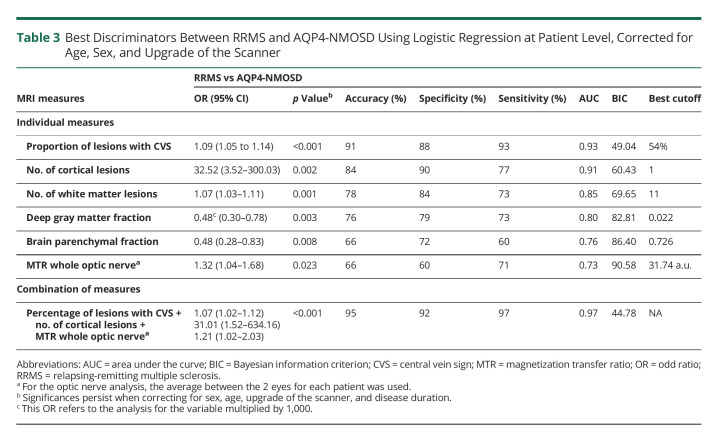
Best Discriminators Between RRMS and AQP4-NMOSD Using Logistic Regression at Patient Level, Corrected for Age, Sex, and Upgrade of the Scanner

#### RRMS vs MOGAD

Brain white matter lesion number was the most accurate MRI measure to predict RRMS rather than MOGAD (OR: 1.89, 95% CI: 1.20–2.99, accuracy: 94%, specificity: 94%, sensitivity: 93%, AUC: 0.99, *p* = 0.006). This means that per each unit of increase in number of lesions, there is an 89% increase in the risk of having RRMS rather than MOGAD. The best cutoff value that predicted RRMS was a number of white matter lesions of 5.

Other measures individually associated with a higher risk of RRMS than MOGAD were a higher number of cortical lesions (OR: 24.68, 95% CI: 2.82–215.65, accuracy: 84%, specificity: 97%, sensitivity: 71%, AUC: 0.87, *p* = 0.004), higher RNFL thickness (OR: 1.06, 95% CI: 1.02–1.12, accuracy: 80%, specificity: 79%, sensitivity: 81%, AUC: 0.83, *p* = 0.009), lower deep gray matter fraction (OR: 0.24, 95% CI: 0.10–0.56, accuracy: 79%, specificity: 71%, sensitivity: 83%, AUC: 0.89, *p* = 0.001), and higher proportion of patients with at least 1 cervical cord lesion (OR: 80.01, 95% CI: 4.03–1,591.84, accuracy: 79%, specificity: 56%, sensitivity: 90%, AUC: 0.86, *p* = 0.004). The combination of higher number of cortical lesions and higher RNFL thickness achieved the highest accuracy in predicting a diagnosis of RRMS rather than MOGAD (accuracy: 84%, specificity: 92%, sensitivity: 77%, AUC: 0.94, *p* < 0.001) (Table 4).

**Table 4:**
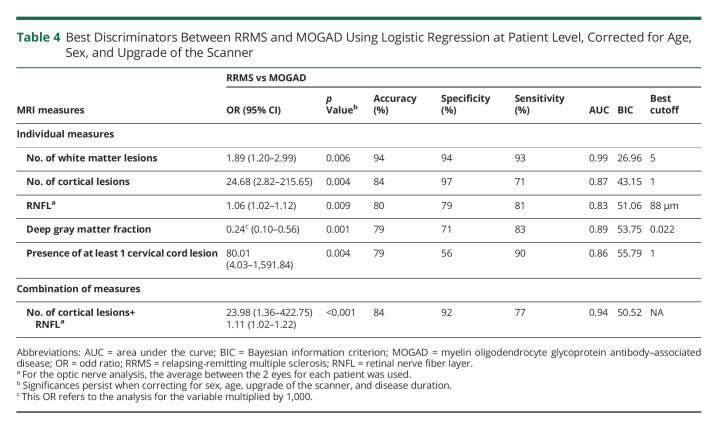
Best Discriminators Between RRMS and MOGAD Using Logistic Regression at Patient Level, Corrected for Age, Sex, and Upgrade of the Scanner

#### AQP4-NMOSD vs MOGAD

The presence of at least 1 cervical cord lesion was the only MRI measure, which predicted AQP4-NMOSD than MOGAD (OR: 30.36, 95% CI: 2.15–427.88, accuracy: 71%, specificity: 65%, sensitivity: 76%, AUC: 0.68, *p* < 0.001).

This study provides Class II evidence that selected conventional and advanced brain, cord, and optic nerve MRI and OCT markers distinguish adult patients with RRMS from APQ4-NMOSD and MOGAD.

## Discussion

In this work, we identified differences in brain, cervical cord, and optic nerve involvement between nonacute RRMS, AQP4-NMOSD, and MOGAD patient groups using different imaging modalities. The key findings are as follows: (1) the number of brain cortical and white matter lesions consistently differentiates RRMS from the 2 antibody-mediated diseases, whereas the CVS best discriminates between RRMS and AQP4-NMOSD; (2) MTR of the optic nerve increases the accuracy in differentiating RRMS from AQP4-NMOSD, whereas RNFL thickness discriminates RRMS from MOGAD; and (3) AQP4-NMOSD and MOGAD share more similarities than differences, and the only imaging marker that distinguished these groups was the presence of at least 1 cervical cord lesion. Our findings may be particularly useful in clinical practice to support a clinical diagnosis and exclude an antibody-mediated condition when the antibody testing is unavailable or suboptimal or when there is a suspicion of false-negative/positive serologic testing results.

The most accurate MRI measure that predicted RRMS rather than AQP4-NMOSD was the proportion of lesions with the CVS (84% vs 33%), extending our previous findings^21^ to the wider spectrum of NMOSD. Of interest, the CVS was detected in 78% of lesions in patients with MOGAD, which is twice as much as in AQP4-NMOSD, but it was not able to distinguish between AQP4-NMOSD and MOGAD; these findings extend the results of a previous pilot study using clinical MRI scans in a smaller number of patients.^22^ A pathologic study has demonstrated that demyelinating plaques in MOGAD may arise around multiple small vessels,^44^ whereas in NMOSD, demyelination is secondary to astrocytic damage, which may occlude the veins, thereby making them undetectable on MRI.^45^

The MRI marker that reached the highest accuracy in separating RRMS from MOGAD was the number of brain white matter lesions, which was also the third most accurate measure that distinguished RRMS from AQP4-NMOSD. In our study, brain MRI lesions were found in a minority of patients with MOGAD (27%), and this can be explained by 2 main factors. First, a sizeable proportion (87%) of patients with MOGAD presented with symptoms suggestive of optic neuritis and myelitis rather than ADEM or focal cortical encephalitis. Second, a complete resolution of brain lesions outside the acute phase is common in MOGAD,^46^ lowering the chance of finding lesions in stable patients. Therefore, our results suggest that in a patient under investigation for a suspected inflammatory demyelinating disorder, a high number of brain white matter lesions points toward a diagnosis of MS rather than MOGAD and AQP4-NMOSD. We did not look at differences in lesion distribution due to the low number of patients with brain lesions. Further studies with larger cohorts are needed to evaluate whether different lesion locations and shapes may help further discriminate the diseases.

The number of cortical lesions was the second most accurate MRI marker indicating a diagnosis of RRMS rather than AQP4-NMOSD or MOGAD. Although cortical demyelination is typical of MS, up to the point that the presence of cortical lesions has been introduced in the last revision of the MS diagnostic criteria,^1^ they are rarely detected in NMOSD.^19^ We extended these investigations to patients with MOGAD by demonstrating that cortical lesions are not seen in nonacute patients. This is in disagreement with a neuropathologic study showing subpial demyelination with cortical involvement in MOGAD, similar to that seen in MS.^44^ This discrepancy may be explained by the limited ability of MRI to detect cortical lesions in vivo, with the most abundant subpial demyelinating remaining unrecognized,^47^ and/or by the different patient characteristics in the studies. In our cohort, patients had adult-onset MOGAD and presented mostly with an NMOSD-like phenotype rather than ADEM, and none presented with focal cortical encephalitis.^44^

Of interest, we demonstrated that higher MTR of the optic nerve increases the accuracy of the CVS and cortical lesions in discriminating RRMS from AQP4-NMOSD, whereas greater RNFL thickness achieved a high accuracy in differentiating RRMS from MOGAD, alone or in combination with cortical lesions. Notably, this study assessed the discriminative role of optic nerve measures at a patient level, whereas the majority of previous studies comparing the sensitivity of OCT and MRI measures mostly focused on the differences between eyes with and without prior optic neuritis,^30^ which may underestimate the effect of subclinical optic nerve involvement occurring in the 3 diseases.^48^

We showed that MTR may be a particularly appropriate nonconventional MRI technique to detect differences between NMOSD and RRMS, using an innovative ROI approach as preprocessing, thus reducing the potential bias introduced by eye motion during the scans. However, this technique remains complex, and validation is crucial before developing clinical applications. Beyond nonconventional MRI, our results further support the role of OCT, which can be easily available in clinic, to objectively demonstrate a differential pattern of optic nerve involvement in the nonacute phases of the 3 diseases.

We found that the 2 antibody-mediated diseases were more similar than different in imaging characteristics, and the only marker differentiating them was the presence of at least 1 cervical cord lesion. This is as expected and reflects the differential involvement of the spinal cord across the 3 diseases.^15^ By contrast, no conventional cord imaging measure contributed to the differentiation between diseases, despite showing different patterns of damage. Further studies looking at different cord segments, including sagittal and axial sequences of the thoracolumbar/conus regions, are needed to accurately quantify the overall extent of cord damage in the 3 diseases.

Unlike cervical cord advanced MRI markers, brain atrophy contributed to discriminate between the diseases with a moderate accuracy, which is consistent with a previous study reporting the power of gray matter measures in differentiating MS from NMO using automatic classification algorithms^49^ and highlight the need for an implementation of methodologies for the translation of atrophy measures in clinical practice, as they may facilitate the discrimination between MS and its mimics.^50^

Finally, although in RRMS and AQP4-NMOSD, brain, spinal cord, and optic nerve imaging measures correlated with disability, in MOGAD, we found associations only when considering optic nerve measures. This may be because the outcome measures we used may be not sufficiently sensitive in MOGAD and do not reflect patients' disabilities. More disease-specific outcome measures to MOGAD, sensitive to different disabilities, are needed.

This study has some limitations. First, the lack of availability of scans at disease onset did not allow us to explore the ability of imaging markers to discriminate the diseases at onset. Although we have adjusted the statistical analysis for disease duration, we studied nonacute patients, not at disease presentation. Further studies are required to evaluate whether these imaging parameters are useful to distinguish patients at onset.

Second, some of the discriminating features are already included in the diagnostic criteria for the diseases (i.e., cortical lesions for MS and optic nerve and cervical cord involvement in NMOSD).^1,33^ We have not identified distinguishing brain features between patients with AQP-NMOSD and MOGAD. Nevertheless, we did identify additional markers to differentiate MS from the 2 Ab-mediated diseases (CVS, atrophy, and optic nerve measures), which should be used as part of the diagnostic criteria. Future studies may investigate the added value of these imaging markers for MS diagnosis, considering also clinical and demographic variables.

Third, the cross-sectional design of the study did not allow us to investigate whether the diseases differ in terms of MRI changes over time. A longitudinal analysis may identify differential patterns of inflammation and neurodegeneration that could better separate these diseases and predict the course of each demyelinating disorder.

In conclusion, the combination of presence of cortical lesions, CVS, and changes in optic nerve markers achieves a high accuracy in distinguishing RRMS from APQ4-NMOSD and MOGAD. When, especially outside the acute phase, serologic testing is unavailable or ambiguous, or a false-negative serologic result is suspected, these markers can be of value to support the differential diagnosis.

## Glossary

9-HPT: 9-hole peg test
Ab: antibody
AQP4-NMOSD: aquaporin-4 antibody–positive neuromyelitis optica spectrum disorder
AUC: area under the curve
CBA: cell-based assay
CSA: cross-sectional area
CVS: central vein sign
DTI: diffusion tensor imaging
EDSS: Expanded Disability Status Scale
GCIPL: ganglion cell–inner plexiform layer
MOGAD: myelin oligodendrocyte glycoprotein antibody–associated disease
MTR: magnetization transfer ratio
OCT: optical coherence tomography
RC: regression coefficient
RRMS: relapsing-remitting multiple sclerosis
SWI: susceptibility-weighted imaging
TWT: timed 25-foot walk test
